# Characterization, evaluation of nutritional parameters of Radix isatidis protein and its antioxidant activity in D-galactose induced ageing mice

**DOI:** 10.1186/s12906-019-2726-y

**Published:** 2019-11-06

**Authors:** Ping Xiao, Hongzhi Huang, Xiang Li, Jianwei Chen, Jin-ao Duan

**Affiliations:** 1 Jiangsu Collaborative Innovation Center of Chinese Medicinal Resources Industrialization, National and Local Collaborative Engineering Center of Chinese Medicinal Resources Industrialization and Formulae Innovative Medicine, Jiangsu Key Laboratory for High Technology Research of TCM Formulae, and State Administration of Traditional Chinese Medicine Key Laboratory of Chinese Medicinal Resources Recycling Utilization, Nanjing University of Chinese Medicine, Nanjing, 210023 China; 2 College of Pharmacy, Nanjing University of Chinese Medicine, Nanjing, 210023 China

**Keywords:** Radix isatidis protein, Oxidative damage, Antioxidant activity, Protein composition, D-galactose

## Abstract

**Background:**

Radix isatidis (*Isatis indigotica* Fort.) is an ancient medicinal herb, which has been applied to the prevention and treatment of influenza virus since ancient times. In recent years, the antioxidant activity of Radix isatidis has been widely concerned by researchers. Our previous studies have shown that Radix isatidis protein (RIP) has good antioxidant activity in vitro. In this study, the composition of the protein was characterized and its antioxidant activity in vivo was evaluated.

**Methods:**

The model of oxidative damage in mice was established by subcutaneous injection of D-galactose for 7 weeks. Commercially available kits were used to determine the content of protein and several oxidation indexes in different tissues of mice. The tissue samples were stained with hematoxylin and eosin (H&E) and the pathological changes were observed by optical microscope. The molecular weight of RIP was analyzed by sodium dodecyl sulfate polyacrylamide gel electrophoresis (SDS-PAGE). The amino acid composition of RIP was determined by a non-derivative method developed by our research group.

**Results:**

RIP significantly increased the activities of antioxidant enzymes such as SOD, CAT, GSH-Px and total antioxidant capability (TAOC) but decreased the MDA level in the serum, kidney and liver. H&E stained sections of liver and kidney revealed D-galactose could cause serious injury and RIP could substantially attenuate the injury. The analysis of SDS-PAGE showed that four bands with molecular weights of 19.2 kDa, 21.5 kDa, 24.8 kDa and 40.0 kDa were the main protein components of RIP.

**Conclusions:**

The results suggested that RIP had excellent antioxidant activity, which could be explored as a health-care product to retard aging and a good source of protein nutrition for human consumption.

## Background

Radix isatidis is the dried root of *Isatis indigotica* Fort. in the Brassicaceae family. It is primarily distributed in Anhui, Hebei, Jiangsu and Gansu provinces of China. Compounds in Radix isatidis mainly include lignans, alkaloids, amino acids, nucleotides, proteins and polysaccharides [1, 2]. As a common Chinese medicine, Radix isatidis has been used in China for more than 2000 years with good clinical effect [3]. In addition to antiviral activity, the antioxidant activity of Radix isatidis is also concerned by researchers [4, 5].

Synthetic antioxidants such as bultylated hydroxyanisole (BHA), butylated hydroxytoluene (BHT) and propyl gallate (PG) have potential hepatotoxicity and carcinogenic effects [6, 7]. These concerns have triggered great interest in the exploration and discovery of natural antioxidants as alternatives to the current practice [8]. Plant protein is an important bioactive substance in traditional Chinese medicine, most of which have antioxidant activity. Therefore, screening proteins with potent antioxidant activity has become a new trend in biology, medicine and food science. Antioxidant peptides could effectively eliminate excess reactive oxygen free radicals in the body and protect the normal structure and function of cells and mitochondria. They were commonly used in the prevention and treatment of free radical-induced diseases and anti-aging. Generally, antioxidant peptides encoded in proteins are released after heat treatment and digestion to play an antioxidant role [9].

Our previous study found that the high polar part of Radix isatidis has good antioxidant activity [10]. Radix isatidis contains a lot of water-soluble protein, accounting for 6~10%. We have identified some of antioxidant peptides from RIP using proteomics techniques (unpublished data). RIP is a kind of non-immune source protein, which can promote the division of thymus T-lymphocyte, improve the immune ability of the body, and have a good therapeutic and preventive effect on influenza [11, 12]. Antioxidants improve health not only by increasing antioxidant activity but also by inducing the protective immune response [13]. Previous researches conducted in our laboratory indicated that the antioxidant activities of Radix isatidis protein hydrolysates were significantly enhanced after simulated gastrointestinal digestion in vitro [14]. However, to our knowledge, it has not yet been reported that RIP had antioxidant activity in vivo. Consequently, it was necessary to study the antioxidant activities in vivo to confirm whether RIP was a good antioxidant.

This study set out to investigate the composition and antioxidant activities of RIP. We reported in detail the antioxidant activities of RIP by using D-galactose induced aging mice as in vivo model. The protein constituents were analyzed by SDS-PAGE and amino acids content of RIP was determined. This study could serve as a reference for the comprehensive utilization of RIP which was previously considered as waste.

## Materials and methods

### Reagents and materials

Radix isatidis was collected from an officially certified standardized planting base in Tongling city of the Anhui province (China). The botanical origin was authenticated by Professor Jianwei Chen (Nanjing University of Chinese Medicine). A voucher specimen has been deposited in the Herbarium of Nanjing University of Chinese Medicine (voucher No. 120615).

Dialysis bags were purchased from Yuanye Biotechnology Co., Ltd. (Shanghai, China). BCA protein assay kits and antioxidant assay kits such as superoxide dismutase (SOD), catalase (CAT), glutathione peroxidase (GSH-Px), total antioxidant capability (T-AOC) and malondialdehyde (MDA) were purchased from Nanjing Jiancheng Bioengineering Institute (Nanjing, China). Vitamin C (VC) and other chemical reagents were bought from Sinopharm Chemical Reagent Co., Ltd. (Shanghai, China). All reagents meet the purity required by the experiment.

### Preparation of Radix isatidis protein

The preparation method of RIP referred to the method we have reported [14]. A certain amount of Radix isatidis powder was added to 20 times (m/v) of 50 mM Tris-HCl solution (pH = 7.8) for full immersion, and ultrasonic extraction was conducted for 60 min. Then the extracted suspension was centrifuged at 3000 g for 5 min. The supernatant was collected and ammonium sulfate was slowly added to a concentration of 70% (m/v). The mixture was placed in a refrigerator at 4 °C overnight. After centrifugation at 3500 g for 10 min the protein precipitant was collected. The protein was re-dissolved with distilled water and then loaded into dialysis bags (molecular mass cut off, 1000 Da) to remove residual salt and small molecule compounds at 4 °C. The dialyzed protein solution was dried by freeze-drying and stored in a refrigerator at − 80 °C for subsequent experiments.

### Analysis of the Radix isatidis protein

#### Sodium dodecyl sulfate polyacrylamide gel electrophoresis (SDS-PAGE) analysis

The RIP sample was analyzed by SDS-PAGE with slight modifications of the method described by Prandi [15]. The RIP sample solution (1 mg/ml) and buffer solution were mixed by equal volume. The buffer solution (pH 6.8) consisted of 1% SDS, 0.005% bromophenol blue, 12.5% glycerol and 2.5% 2-mercaptoethanol. Then the mixed solution was placed in a 95 °C water bath for 5 min. After cooling down, prepared samples were separated by precast 12% gel on a Mini Protean II Dual Slab Cell (BIO-RAD) device. The separated gel was stained by Coomassie Brilliant blue method.

#### Amino acid analysis

The RIP sample (5 mg) was hydrolyzed in a sealed tube with 5 ml 6 mM HCl at 110 °C for 24 h. Alkaline hydrolysis was used to determine the tryptophan content. The hydrolyzed sample was centrifuged at 12000 g for 10 min and the supernatant was passed through a 0.22 μm microporous membrane for subsequent analysis. Amino acid composition of RIP was analyzed using the method we developed previously [16].

#### Evaluation of nutritional parameters

The nutritional value of RIP was evaluated by the following formulas [17].

- The proportion of essential amino acids (E) to total amino acids (T) of the protein:

$$ \frac{E}{T}\%=\left(\frac{I+L+K+M+C+F+Y+T+V+H+W}{A+D+R+G+E+I+L+K+M+C+F+Y+P+S+T+V+H+W}\right)\times 100 $$

(b) Amino acid score:

$$ =\left(\frac{\begin{array}{l}\kern1.5em \\ {}\ \mathrm{mg}\ \mathrm{of}\ \mathrm{amino}\ \mathrm{per}\ \mathrm{g}\ \mathrm{test}\ \mathrm{protein}\end{array}}{\mathrm{mg}\ \mathrm{of}\ \mathrm{amino}\ \mathrm{acid}\ \mathrm{per}\ \mathrm{g}\ \mathrm{of}\ \mathrm{FAO}/\mathrm{WHO}\ \mathrm{standard}\ \mathrm{pattern}}\right)\times 100 $$

### Determination of the antioxidant activity of RIP in vivo

#### Animal selection and experimental design

ICR male outbred mice (8-weeks-old) weighing 20.0 ± 2.0 g were purchased from the Jiangsu University of Laboratory Animal Research Center. These mice were randomly divided into six groups with 10 mice in each group after 7-day adaptation period. Mice in the normal control group were given physiological saline solution (0.9% NaCl, w/v) (10 ml/kg bw) per day by subcutaneous injection and gastric gavage. Mice in the model control group were injected subcutaneously with D-galactose (800 mg/kg bw) and given 0.9% NaCl (10 ml/kg bw) by gastric gavage each day. The D-galactose was dissolved in physiological saline solution (0.9%, w/v) at a concentration of 80 mg/ml. The volume of hypodermic injection was 0.1 ml/10 g bw. Mice in the positive control group were treated with VC (100 mg/ kg bw) dissolved in physiological saline by gastric gavage and D-galactose (800 mg/kg bw) by subcutaneous injection per day. Mice in the low dose, medium dose and high dose group were fed with RIP at doses of 25, 50 and 100 mg/kg bw respectively by gastric gavage and subcutaneously injected with D-galactose (800 mg/kg bw) per day. The whole experiment lasted for 7 weeks, and the weight of mice on the first day and every week was recorded.

#### Sample preparation of serum, kidney, liver and brain tissue

After the last drug administration, the mice were fasted for one night and then humanely sacrificed by cervical dislocation. Blood samples of mice were collected immediately from retrobulbar venous plexus and kept at 37 °C for 1 h. Then blood samples were centrifuged at 4000 g for 10 min at 4 °C, and the supernatant was collected as serum for assays. Different tissues were excised and weighed immediately, then homogenized with ice-cold normal saline (0.9%) (0.1 g tissue/ml solution). The 10% homogenate was centrifuged at 4000 g for 20 min at 4 °C and the supernatant was used for further biochemical analysis.

### Histological analysis

The liver and kidney tissues were fixed with fresh solution of 4% paraformaldehyde (pH 7.4) which were prepared for histological analysis. All tissue sections were stained with hematoxylin and eosin (H&E) and viewed under a light microscopy (Axioskop 40, Zeiss, Germany). Pathology reports were issued by a board certified veterinary pathologist who knew nothing about the grouping of our experiment.

### Analysis of kidney, liver, thymus and spleen indices

Different tissues of the mice were thoroughly separated and weighed to calculate the organ index. The organ index was calculated according to the following formula:

$$ \mathrm{Index}=\left(\mathrm{weight}\ \mathrm{of}\ \mathrm{organ}/\mathrm{body}\ \mathrm{weight}\right)\times 1000 $$

### Biochemical analysis

Protein content, activities of catalase (CAT), superoxide dismutase (SOD) and glutathione peroxidase (GSH-Px) and the level of malondialdehyde (MDA) in tissue homogenates and serum samples were tested with commercial kits according to relevant operation instructions.

### Statistical analysis

Data were presented as means ± standard deviation (SD) and sample size is at least 3 in each group. Statistical significance was determined by one-way analysis of variance (ANOVA) followed by Duncan’s multiple range test at a 5% level. If *P* < 0.05, there was significant difference between the two groups.

## Results

### SDS-PAGE analysis

**Fig. 1:**
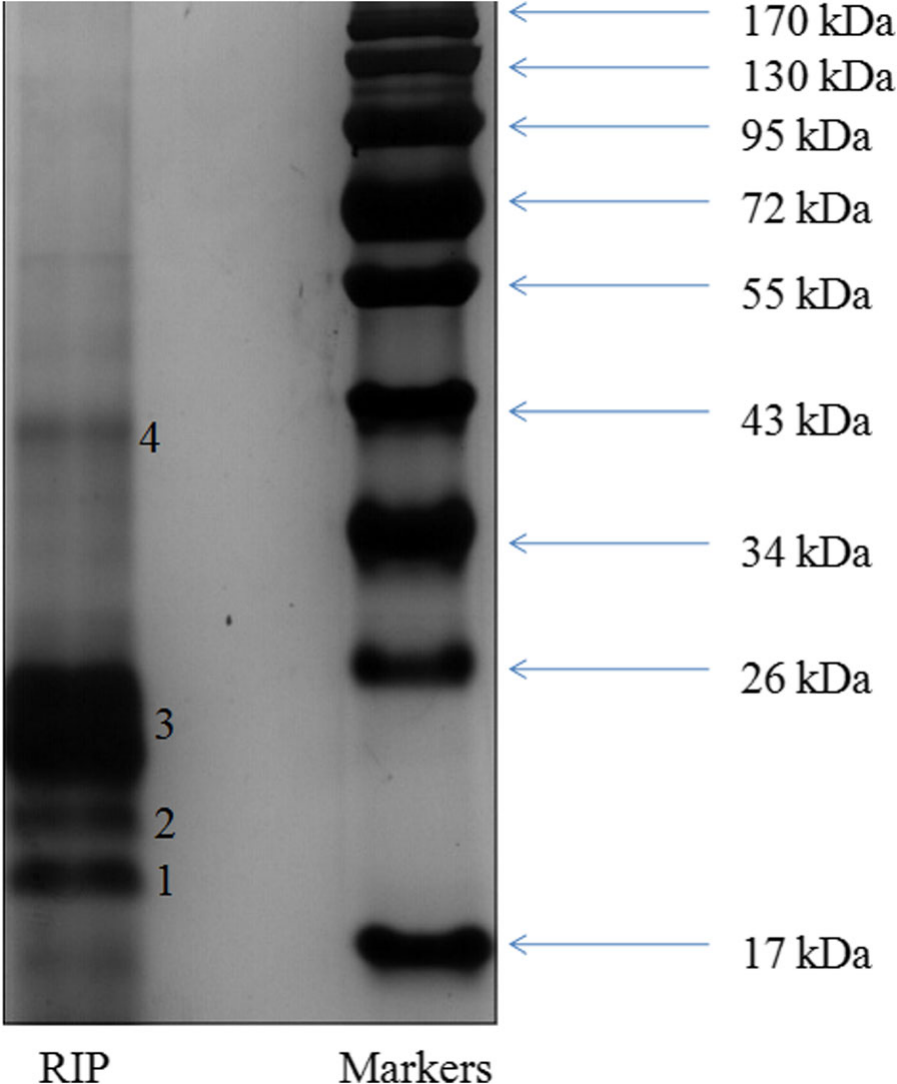
SDS-PAGE profiles of Radix isatidis protein (RIP)

The results of SDS-PAGE were shown in Fig. 1. The molecular weight of RIP was calculated by comparison with standard protein markers. Two bands with molecular weights of 19.2 kDa (1) and 21.5 kDa (2) appeared in the lower portion of the gel. One weak band (4) with an approximate molecular weight of 40.0 kDa was also observed. A band with a molecular weight of 24.8 kDa (3) appeared as the largest band on the gel indicating that it was the most abundant protein in RIP.

### The amino acids composition of RIP

The results of amino acid content of RIP were presented in Table 1. RIP contained a high amount of aspartic acid (9.78 g/100 g of RIP), followed by proline, isoleucine, glutamic acid, glycine, threonine in decreasing amounts. The total values of aromatic amino acids and essential amino acids content met the requirements set by FAO/WHO [18]. The individual content for each of the essential amino acids (isoleucine, threonine, tryptophan and valine) also reached the minimum FAO requirements. The proportion of essential amino acids to the total amino acids (E/T) for RIP was 46.77% and the amino acid score was 101.33, suggesting that the RIP may have good nutritional value.

**Table 1:** Amino acid composition of RIP and the FAO/WHO suggested requirements (2–5 years old) for the essential amino acids (g/100 g of protein)

Amino acids	RIP	FAO/WHO suggested requirements (2–5 years old)
Isoleucine	7.36 ± 0.51	2.8
Leucine	3.92 ± 0.24	6.6
Lysine	0.46 ± 0.06	5.8
Cysteine	0.01 ± 0.00	
Methionine	0.11 ± 0.02	
Total sulphur amino acids	0.12	2.5
Tyrosine	0.85 ± 0.06	
Phenylalanine	4.08 ± 0.19	
Total aromatic amino acids	4.93	6.3
Threonine	5.06 ± 0.83	3.4
Tryptophan	2.57 ± 0.44	1.1
Valine	7.62 ± 0.08	3.5
Histidine	1.74 ± 0.04	1.9
Total essential amino acids	33.79	32.8
Arginine	2.95 ± 0.24	
Aspartic acid + asparagine	9.78 ± 0.65	
Glutamic acid + glutamine	6.03 ± 0.42	
Serine	2.36 ± 0.04	
Proline	8.88 ± 0.49	
Glycine	6.03 ± 0.70	
Alanine	2.43 ± 0.21	
Total non-essential amino acids	38.46	
^a^E/T,%	46.77	
Amino acid score	101.33	
^b^Antioxidant amino acids	29.63	

^a^ The proportion of essential amino acids to the total amino acids

^b^Antioxidant amino acids, it contains hydrophobic amino acids (valine, alanine, proline and leucine), aromatic amino acids (tyrosine, histidine, and phenylanine), and methionine [19]

### Body weight

At the beginning of the experiment, there were no remarkable difference among the mean body weight of the ICR mice in all groups (*P* > 0.05) (Table 2). The mean body weight of the mice in all the groups, except the model control group, increased for the duration of the experiment. During the 7 weeks of the experiment, the mean body weight of animals in the model control group was significantly lower than the normal control group and the other treatment groups (*P* < 0.05). However, there were no significant differences between the normal control group and other treatment groups.

**Table 2:** Effects of increasing age and different treatments on the body weights (g) of ICR mice

Group	Week							
	0	1	2	3	4	5	6	7
Normal control	21.26 ± 1.18	26.60 ± 1.57	29.50 ± 1.02	32.32 ± 1.30	36.33 ± 1.25	38.34 ± 1.57	40.08 ± 1.60	42.77 ± 1.15
Model control	21.49 ± 1.24	26.59 ± 2.62	29.74 ± 1.28	32.40 ± 1.14	36.51 ± 1.32	35.68 ± 1.65^d^	35.04 ± 0.92^d^	34.19 ± 1.16^d^
Positive control	21.38 ± 1.05	26.77 ± 1.11	28.41 ± 1.67	32.33 ± 2.23	35.99 ± 2.62	36.54 ± 1.59^ac^	39.02 ± 2.39^b^	41.59 ± 2.92^b^
RIP (25 mg/kg)	21.26 ± 0.75	26.08 ± 1.43	27.67 ± 1.24^ac^	31.08 ± 1.57	33.86 ± 2.47^ac^	36.14 ± 2.42^ac^	38.00 ± 2.84^bc^	40.65 ± 1.25^b^
RIP (50 mg/kg)	21.56 ± 0.69	26.26 ± 0.85	30.85 ± 1.27	32.18 ± 0.84	35.54 ± 2.38	37.83 ± 2.89^c^	38.63 ± 3.06^bc^	40.12 ± 3.01^b^
RIP(100 mg/kg)	21.12 ± 1.43	26.82 ± 1.09	31.16 ± 1.24	33.73 ± 1.91	36.79 ± 1.57	37.81 ± 2.47^c^	38.27 ± 2.98^bc^	41.12 ± 2.16^b^

Results are given as mean body weights ± standard deviation (S.D.) (*n* = 10) and evaluated by one-way ANOVA followed by the Duncan’s multiple-range tests. Differences were considered to be statistically significant if *P* < 0.05

^a^ *P* < 0.05, compared with model group

^b^ *P* < 0.01, compared with model group

^c^ *P* < 0.05, compared with normal group

^d^ *P* < 0.01, compared with normal group

### The effect of the RIP on kidney, liver, thymus and spleen indexes in aging mice

As shown in Table 3, the kidney, liver, thymus and spleen indexes in the model control group were significantly lower than the normal control group and other treatment groups (*P* < 0.05). Administration of RIP and VC seemed to have remarkable effects on increasing the indexes of the four organs in a dose-dependent manner.

**Table 3:** Effects of different treatments on the liver, kidney, thymus and spleen indices in aging mice

Index (mg/g·bw)	Group					
	Normal control	Model control	Positive control	RIP (25 mg/kg)	RIP (50 mg/kg)	RIP (100 mg/kg)
liver	42.27 ± 2.72	37.93 ± 3.49^d^	40.43 ± 2.03^ac^	38.59 ± 3.14^ac^	40.54 ± 3.40^bc^	40.72 ± 2.48^bc^
kidney	15.59 ± 1.46	13.74 ± 1.69^d^	14.04 ± 1.41^ac^	13.97 ± 1.20^c^	14.39 ± 1.88^ac^	14.76 ± 1.49^ac^
spleen	2.45 ± 1.30	2.17 ± 0.50^c^	2.25 ± 0.75^a^	2.29 ± 0.67^ac^	2.30 ± 1.01^ac^	2.37 ± 0.58^a^
thymus	2.48 ± 0.64	2.02 ± 0.87^c^	2.32 ± 0.50^a^	2.28 ± 0.34^ac^	2.35 ± 0.50^a^	2.36 ± 0.34^a^

Data were presented as means ± S.D. (*n* = 10) and evaluated by one-way ANOVA followed by the Duncan’s multiple-range tests

^a^ *P* < 0.05, compared with model group

^b^ *P* < 0.01, compared with model group

^c^ *P* < 0.05, compared with normal group

^d^ *P* < 0.01, compared with normal group

### Histopathological observation

#### The effect of RIP on histological changes in livers

**Fig. 2:**
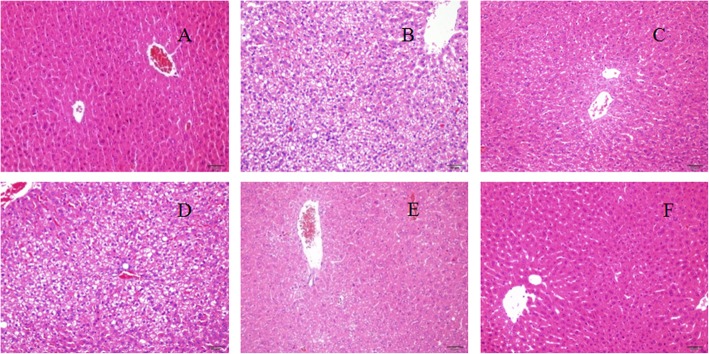
Histopathological observation of liver in aging mice. Magnification, × 200: **a** normal control group; (**b**) model control group; (**c**) positive control group; (**d**) RIP of low dose group; (**e**) RIP of medium dose group; (**f**) RIP of high dose group

Histologic examinations of liver sections of aging mice treated with VC and different doses of RIP were illustrated in Fig. 2. Compared with the normal group (Fig. 2a), the hepatocytes in the model group (Fig. 2b) had edema and partial necrosis. A large number of vacuolar changes were observed in the liver tissues of the mice in the model group, indicating obvious looseness, swelling, focal necrosis, and inflammatory cell infiltration. However, there was significant improvement in the high-dose group (100 mg/kg bw) and the medium-dose group (50 mg/kg bw), but not in the low-dose group (25 mg/kg bw). The results of histopathological evaluation showed that RIP exhibited a hepatoprotective effect against D-galactose-induced liver injury.

#### The effects of RIP on histology changes in kidneys

**Fig. 3:**
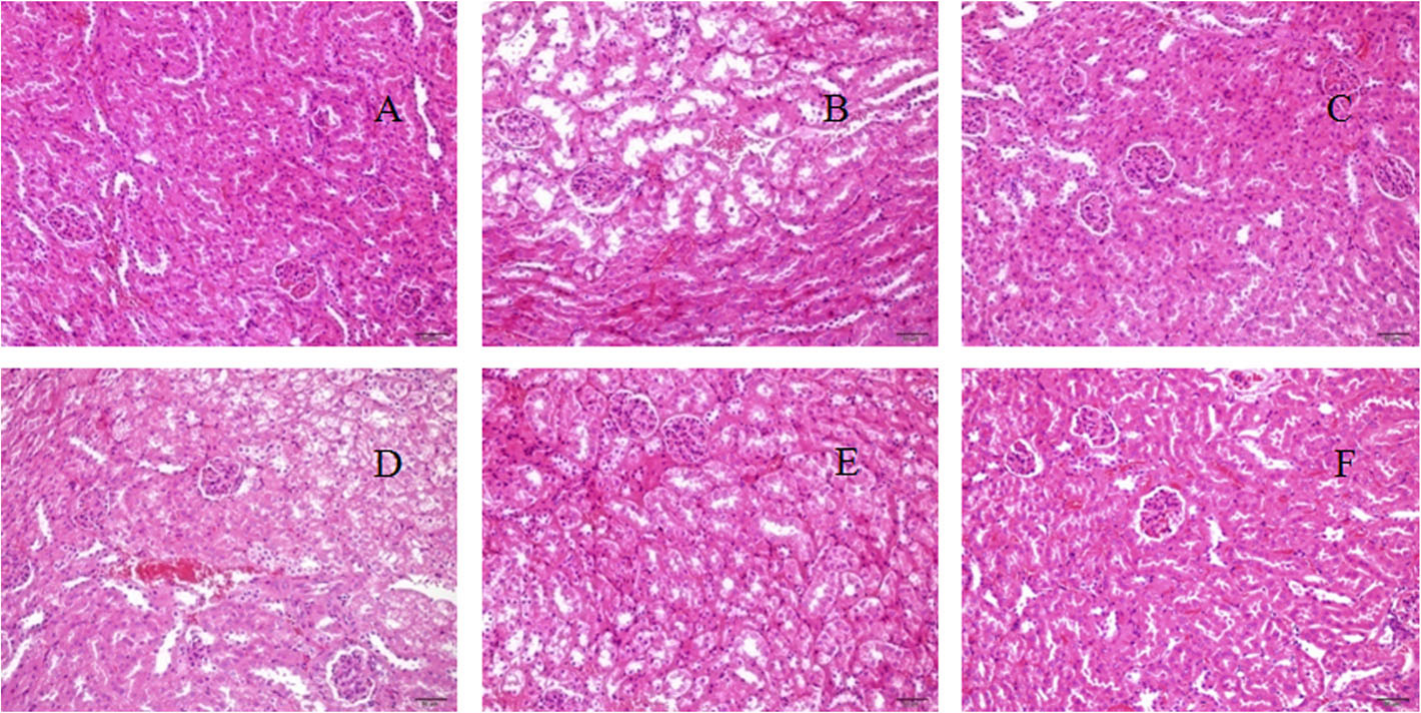
Histopathological observation of kidney in aging mice. Magnification, × 200: **a** normal control group; (**b**) model control group; (**c**) positive control group; (**d**) RIP of low dose group; (**e**) RIP of medium dose group; (**f**) RIP of high dose group

The degree of renal injury of D-galactose was observed by pathological section. It could be seen from Fig. 3 that different concentrations of RIP have certain protective effects on renal damage caused by D-galactose. The mice in the model group had significant pathological changes in their kidneys with distal convoluted tubule expansion and vacuoles in epithelial cells (Fig. 3b). These alterations in the kidneys of aging mice were ameliorated almost to normal levels after treatments with high doses of RIP, shown in Fig. 3f. Hence, RIP could alleviated kidney damage induced by D-galactose.

#### The effect of the RIP on the activities of antioxidant enzymes in liver, kidney and brain in aging mice

The effects of RIP on activities of various antioxidant enzymes in serum, livers and kidneys were presented in Tables 4, 5, 6 respectively. Compared with the normal control group, MDA concentration in serum and tissue of mice in model control group increased significantly (*P* < 0.05), but antioxidant enzymes (SOD, CAT, GSH-Px and TAOC) activity decreased significantly. Different doses of RIP (25~100 mg/kg) significantly (*P* < 0.05) inhibited the formation of MDA and increased the activities of antioxidant enzymes in serum, livers and kidneys. As shown in Table 7, RIP does not significantly protect the brain from oxidative damage (*P* > 0.05).

**Table 4:** Effects of different treatments on the activities of CAT (U/ml), SOD(U/ml), GSH-Px (U/ml) and levels of MDA (nmol/ml) and TAOC (U/ml) in the serum of aging mice

Group	MDA	TAOC	CAT	SOD	GSH-Px
Normal control	16.93 ± 0.63	8.09 ± 0.42	29.01 ± 1.79	208.63 ± 27.29	1094.56 ± 69.89
Model control	24.87 ± 0.89^d^	6.52 ± 0.48^d^	14.00 ± 1.74^d^	157.25 ± 34.62^d^	784.50 ± 44.70^d^
Positive control	19.77 ± 0.58^ad^	8.24 ± 0.62^b^	27.78 ± 1.53^b^	207.49 ± 32.40^b^	1069.13 ± 24.48^b^
RIP (25 mg/kg)	24.58 ± 0.39^d^	7.15 ± 0.63^c^	16.32 ± 2.33^ad^	165.53 ± 18.84^ad^	874.43 ± 31.82^ad^
RIP (50 mg/kg)	23.32 ± 0.52^ad^	7.65 ± 0.35^a^	24.83 ± 2.13^bc^	187.74 ± 13.60^bd^	950.97 ± 19.70^bc^
RIP (100 mg/kg)	20.99 ± 0.62^bd^	8.58 ± 0.53^b^	26.49 ± 1.33^bc^	227.47 ± 16.31^bc^	1167.18 ± 82.58^b^

Data were presented as means ± S.D. (n = 10) and evaluated by one-way ANOVA followed by the Duncan’s multiple-range tests

^a^ *P* < 0.05, compared with model group

^b^ *P* < 0.01, compared with model group

^c^ *P* < 0.05, compared with normal group

^d^ *P* < 0.01, compared with normal group

**Table 5:** Effects of different treatments on the activities of CAT (U/mg protein), SOD (U/mg protein), GSH-Px (U/mg protein) and levels of MDA (nmol/mg protein) and TAOC (U/mg protein) in the livers of aging mice

Group	MDA	TAOC	CAT	SOD	GSH-Px
Normal control	1.43 ± 0.10	1.22 ± 0.15	36.18 ± 0.30	171.39 ± 16.44	399.67 ± 16.92
Model control	2.78 ± 0.09^d^	0.96 ± 0.11^c^	28.05 ± 3.28^d^	100.89 ± 3.77^d^	343.89 ± 19.44^a^
Positive control	1.34 ± 0.09^b^	2.08 ± 0.33^bd^	49.67 ± 2.24^bd^	116.71 ± 8.18^ad^	479.49 ± 23.56^bd^
RIP (25 mg/kg)	2.22 ± 0.14^ad^	1.09 ± 0.14^c^	35.51 ± 1.34^b^	107.36 ± 10.86^d^	345.65 ± 12.35^c^
RIP (50 mg/kg)	2.01 ± 0.10^ab^	1.59 ± 0.17^ac^	39.41 ± 1.97^bc^	124.62 ± 11.53^ad^	378.94 ± 15.05^a^
RIP (100 mg/kg)	1.80 ± 0.10^ac^	1.78 ± 0.22^bc^	42.57 ± 2.19^bd^	136.92 ± 9.15^bd^	414.85 ± 19.95^dc^

Data were presented as means ± S.D. (*n* = 10) and evaluated by one-way ANOVA followed by the Duncan’s multiple-range tests

^a^ *P* < 0.05, compared with model group

^b^ *P* < 0.01, compared with model group

^c^ *P* < 0.05, compared with normal group

^d^ *P* < 0.01, compared with normal group

**Table 6:** Effects of different treatments on the activities of CAT (U/mg protein), SOD (U/mg protein), GSH-Px (U/mg protein) and levels of MDA (nmol/mg protein) and TAOC (U/mg protein) in the kidneys of aging mice

Group	MDA	TAOC	CAT	SOD	GSH-Px
Normal control	1.46 ± 0.24	1.69 ± 0.10	32.77 ± 2.44	102.35 ± 5.42	248.16 ± 8.14
Model control	2.38 ± 0.26^d^	0.88 ± 0.08^d^	25.44 ± 1.78^d^	74.33 ± 6.96^d^	174.23 ± 7.92^d^
Positive control	1.17 ± 0.28^bc^	1.61 ± 0.12^b^	34.99 ± 0.92^b^	87.27 ± 8.00^ad^	259.39 ± 10.37^bc^
RIP (25 mg/kg)	1.84 ± 0.38^ac^	1.28 ± 0.12^ac^	26.36 ± 1.72^d^	76.00 ± 9.04^d^	186.41 ± 8.45^ad^
RIP (50 mg/kg)	1.67 ± 0.29^a^	1.42 ± 0.16^bc^	32.62 ± 1.89^a^	92.10 ± 4.59^bc^	212.57 ± 6.82^ac^
RIP (100 mg/kg)	1.30 ± 0.35^a^	1.81 ± 0.28^b^	43.92 ± 0.87^bd^	112.48 ± 6.13^bc^	236.78 ± 9.15^bc^

Data were presented as means ± S.D. (*n* = 10) and evaluated by one-way ANOVA followed by the Duncan’s multiple-range tests

^a^ *P* < 0.05, compared with model group

^b^ *P* < 0.01, compared with model group

^c^ *P* < 0.05, compared with normal group

^d^ *P* < 0.01, compared with normal group

**Table 7:** Effects of different treatments on the activities of CAT (U/mg protein), SOD (U/mg protein), GSH-Px (U/mg protein) and levels of MDA (nmol/mg protein) and TAOC (U/mg protein) in the brains of aging mice

Group	MDA	TAOC	CAT	SOD	GSH-Px
Normal control	1.07 ± 0.07	1.01 ± 0.15	32.07 ± 2.02	258.22 ± 12.68	319.32 ± 8.94
Model control	1.63 ± 0.27^d^	0.64 ± 0.09^d^	25.75 ± 2.23^d^	162.78 ± 23.86^d^	228.75 ± 6.95^d^
Positive control	0.89 ± 0.08^bc^	1.27 ± 0.12^bc^	49.67 ± 2.24^bd^	250.47 ± 11.65^b^	354.29 ± 11.28^bc^
RIP (25 mg/kg)	1.61 ± 0.07	0.66 ± 0.04	24.27 ± 2.53	164.91 ± 17.81	224.87 ± 12.62
RIP (50 mg/kg)	1.58 ± 0.10	0.67 ± 0.08	25.33 ± 3.19	165.98 ± 15.02	227.56 ± 14.89
RIP (100 mg/kg)	1.55 ± 0.10	0.71 ± 0.20	25.40 ± 2.56	165.47 ± 11.65	229.36 ± 15.63

Data were presented as means ± S.D. (*n* = 10) and evaluated by one-way ANOVA followed by the Duncan’s multiple-range tests

^a^ *P* < 0.05, compared with model group

^b^ *P* < 0.01, compared with model group

^c^ *P* < 0.05, compared with normal group

^d^ *P* < 0.01, compared with normal group

## Discussion

Protein is another important biological active component in plants besides alkaloids, polysaccharides and saponins, which has attracted more and more attention in recent years. Some studies have found that plant proteins can directly remove free radicals and play an anti-oxidation role. At present, plant-derived antioxidant proteins have unique physiological functions and broad application prospects because of their superior antioxidant activity and high safety. The antioxidant activity of RIP was affected by different kinds of amino acids in different molecular weight peptide segments. According to our current research results, the molecular weight of RIP is mainly distributed in 17~26 kDa. RIP was rich in essential amino acids and could be used as a nutritional supplement. Antioxidant amino acids might control antioxidant potentials of protein and peptides. According to the literature antioxidant amino acids usually contain hydrophobic amino acids (valine, alanine, proline and leucine), aromatic amino acids (tyrosine, histidine, and phenylanine), and methionine [19]. The total content of antioxidant amino acids in RIP was 29.63 g/100 g. Antioxidant amino acids were implicated in the control of antioxidant potentials of proteins and peptides [20]. It could be inferred that the antioxidant amino acids play an important role in the antioxidant effect of RIP.

Modern medical research shows that immunity is a factor closely related to aging and immune function decline is one of the most important causes of aging [21]. The thymus and spleen are important immune organs, so the thymus and spleen indexes suggest activation, enhancement or strengthening of the immune status or immune function. The results indicated that RIP improved immunity and protected the liver and kidney from oxidative damage in aging mice.

The occurrence of many diseases is related to lipid peroxidation induced by free radicals. Free radicals can induce lipid peroxidation, and one of the most important ways to damage the body is to produce lipid peroxides. Normally, free radicals can maintain a balance below the damage threshold, which depends on the body’s antioxidant system. Under the action of galactose oxidase, D-galactose generates acetaldehyde sugar and hydrogen peroxide, which increase the amount of reactive oxygen and hyperlipidemia, and generates superoxide anion free radicals, thus leading to the aging of the body [22]. MDA is a kind of harmful substance, which can cause the cross-linking polymerization of proteins, nucleic acids and other life macromolecules, and has cytotoxicity. The content of MDA often reflects the degree of lipid peroxidation in vivo, and indirectly reflects the degree of cell damage [23]. Medium and high doses of RIP could significantly reduce the content of MDA in serum and tissues of mice with oxidative damage (*P* < 0.05). This may be due to the effect of RIP on stabilizing the cell membrane structure and inhibiting lipid peroxidation. SOD, GSH-Px, T-AOC and CAT are important antioxidant enzymes in the body, and their activity can comprehensively reflect the antioxidant capacity of the body. With the increase of age, the activity of antioxidant enzymes in human body decreases gradually, mainly because the genes encoding antioxidant enzymes and antioxidant enzymes themselves are damaged by free radical oxidation [24]. RIP could enhance the activity of SOD, GSH-Px, T-AOC and CAT in serum, liver and kidney of mice to enhance the ability of enzymatic system to resist free radical reaction, and reduce the oxidation of free radicals to unsaturated fatty acids, thus reducing the content of MDA to a certain extent. This study showed that RIP could eliminate the damage of free radicals in the body and has a good antioxidant effect in vivo.

## Conclusions

The total content of essential amino acids in RIP met the requirements of FAO/WHO for 2–5 years old infants. RIP was rich in antioxidant amino acids (29.63 g/100 g RIP) which may contribute to the antioxidant activities. Thus, RIP may be suitable for human consumption as a source of protein nutrition and antioxidants. This study firstly evaluated the antioxidant activity of RIP in a D-galactose induced aging mice model in vivo. These results revealed that RIP effectively alleviated the oxidative damage and could be explored as a potential source of protein nutrition for dietary supplement.

## Data Availability

The raw data generated and/or analyzed during the current study are available from the corresponding author on reasonable request.

## Abbreviations

CAT: catalase
FAO: Food and Agriculture Organization
GSH-Px: glutathione peroxidase
H&E: hematoxylin and eosin
HILIC-UPLC-MS/MS: hydrophilic interaction ultra-high-performance liquid chromatography coupled with triple-quadrupole mass spectrometry
MDA: malondialdehyde
RIP: Radix isatidis protein
SDS-PAGE: sodium dodecyl sulfate polyacrylamide gel electrophoresis
SOD: superoxide dismutase
TAOC: total antioxidant capability
